# Airway Obstruction With Severe Hypoxemia Caused by Malformation of an Endotracheal Tube Cuff: A Case Report

**DOI:** 10.1002/ccr3.73724

**Published:** 2026-11-05

**Authors:** Xin Li, Yuwen Chen, Dan Zhou

**Affiliations:** ^1^ Department of Pain Management Deyang People's Hospital Deyang China; ^2^ Department of Pain Management Sichuan Science City Hospital Mianyang China

**Keywords:** airway obstruction, cuff malformation, endotracheal tube, hypoxemia

## Abstract

Airway obstruction and severe hypoxemia directly attributable to an ETT cuff malformation are exceedingly rare. It emphasizes that pre‐use cuff inspection should not be limited to leak testing but should also include an assessment of the cuff's morphological integrity.

## 1 Introduction

Airway management constitutes a core responsibility of anesthesiologists, and endotracheal intubation remains the most secure and effective modality for securing the airway during general anesthesia [1, 2]. The endotracheal tube cuff (ETT) is vital for ensuring adequate ventilation and preventing aspiration; abnormalities can lead to severe airway complications. Previous literature has primarily focused on cuff leaks as a cause of hypoventilation [3, 4, 5], whereas cuff morphological anomalies have received relatively little attention. This case report describes a rare instance of airway obstruction and severe hypoxemia secondary to ETT cuff malformation, analyzes the underlying etiology, and summarizes management strategies to improve clinical awareness of such events.

## 2 Case Presentation/Examination

A 45‐year‐old female (height: 162 cm; weight: 56 kg) scheduled for functional endoscopic sinus surgery under general anesthesia due to chronic sinusitis presented with unremarkable preoperative investigations. The patient was previously healthy, with no history of coronary artery disease, asthma, chronic obstructive pulmonary disease, or other conditions. There was no known allergy to food or medications. The patient was assessed as American Society of Anesthesiologists (ASA) physical status Class I, with a Mallampati Class II airway. Upon arrival in the operating room, peripheral intravenous access was established, and standard monitoring (electrocardiography, non‐invasive blood pressure, and SpO_2_) was initiated. Pre‐anesthetic checks confirmed no leaks in the anesthesia machine or the ETT cuff. General anesthesia was induced with midazolam (2 mg IV), propofol (100 mg IV), cisatracurium besylate (10 mg IV), and sufentanil (25 μg IV). Orotracheal intubation was performed uneventfully under direct videolaryngoscopy using a reinforced ETT (internal diameter: 7.0 mm), with the tube positioned at a depth of 22 cm from the lips. Following cuff inflation, the ETT was connected to the anesthesia circuit; manual ventilation confirmed a normal end‐tidal carbon dioxide (P_ET_CO_2_) waveform, and the tube was secured in place. Mechanical ventilation was initiated in volume‐controlled mode, with the following settings: tidal volume, 450 mL; respiratory rate, 11 breaths per minute; and inspiration‐to‐expiration ratio, 1:2. Initial airway pressure was 13 cm H_2_O and P_ET_CO_2_ was 41 mmHg.

## 3 Methods

Following patient positioning by the surgical team (shoulder roll placement and head fixation) and routine surgical draping, the anesthesia machine alarm activated, indicating elevated airway pressure (35 cm H_2_O). Manual ventilation confirmed high resistance. The ETT position and depth were verified as correct, and the circuit tubing was checked for kinks. Auscultation revealed markedly diminished or absent breath sounds. Interventions included intravenous methylprednisolone (40 mg) and aminophylline (125 mg), yielding no improvement. SpO_2_ then began to decline, and manual ventilation only achieved minimal tidal volumes (approximately 100 mL). Tracheal suctioning retrieved negligible secretions and did not result in clinical improvement, with SpO_2_ further decreasing to 93%. An additional propofol bolus (50 mg IV) and cisatracurium (5 mg IV) were administered concurrently with urgent fiberoptic bronchoscopy. Bronchoscopic visualization was initially obscured by reflected light from secretions. Advancing the scope encountered mild resistance but the carina was reached; both main bronchi appeared patent without obstruction from masses or mucous plugs. After scope withdrawal, airway pressure increased drastically, ventilation became nearly impossible, and SpO_2_ rapidly plummeted to 80%. A critical decision regarding ETT removal was debated. Repeat auscultation confirmed absent breath sounds, manual ventilation was extremely difficult, and SpO_2_ dropped to approximately 60% with evident cyanosis. The decision was made to remove the ETT and institute mask ventilation. Mask ventilation immediately normalized airway pressure, produced adequate chest rise, and SpO_2_ recovered rapidly. Re‐intubation was performed uneventfully, surgery proceeded normally, and the patient recovered fully postoperatively with no sequelae at follow‐up. Examination of the removed ETT after cuff inflation revealed a significant anterior deformity compared to a normal ETT cuff (Figure 1).

**FIGURE 1:**
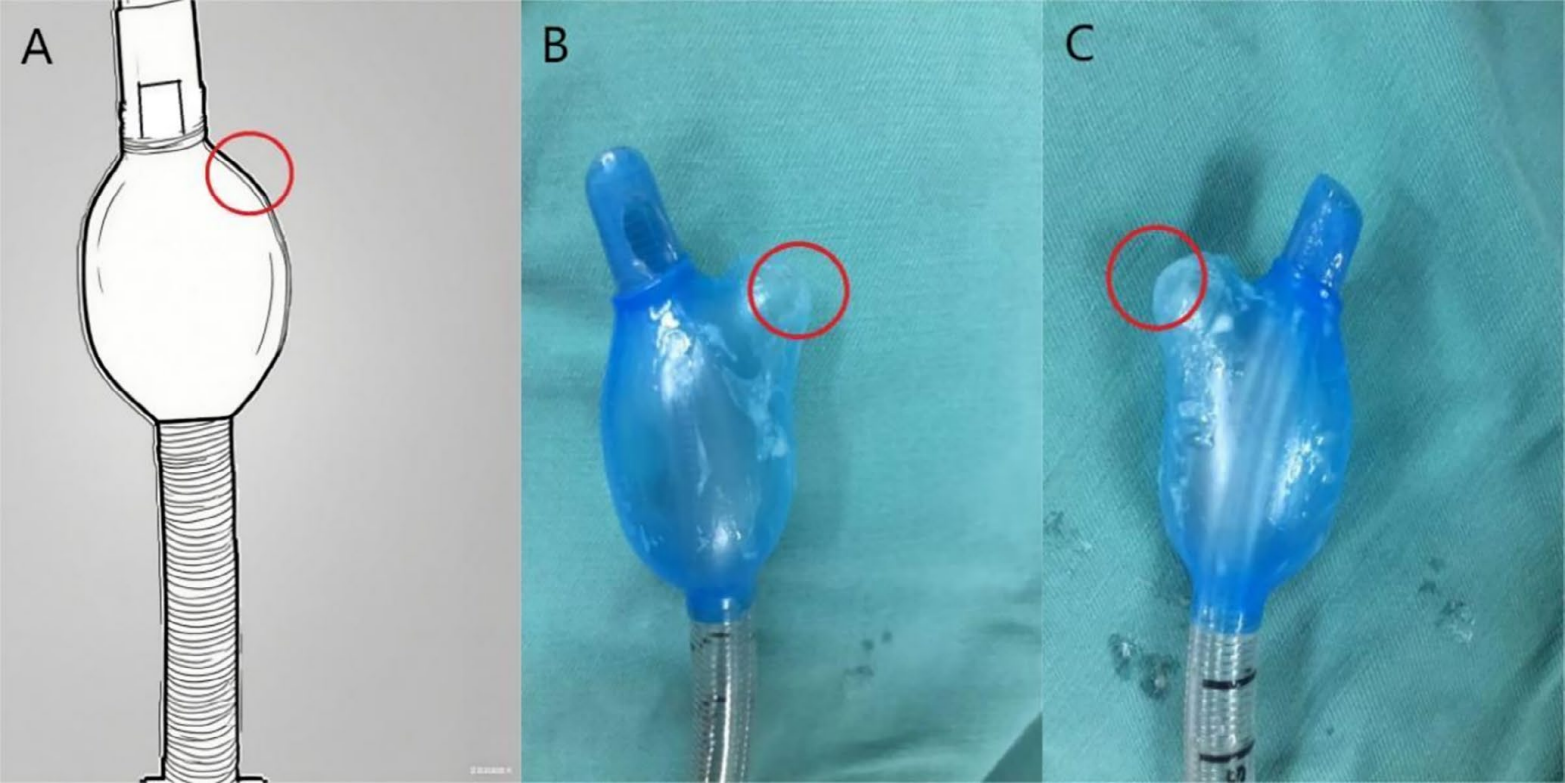
(A) Schematic diagram of a normal endotracheal tube cuff; (B,C) malformed endotracheal tube cuffs (indicated by red circles).

## 4 Conclusion and Results

We report a rare case of severe airway obstruction and hypoxemia caused by a malformed endotracheal tube cuff. Fortunately, following reintubation, all conditions stabilized. The procedure was completed successfully with the patient awake at extubation, and follow‐up was uneventful. This case underscores the necessity of incorporating cuff morphological assessment into the standard pre‐anesthesia checklist, alongside routine leak testing. It also offers insights into the recognition and management of critical intraoperative airway emergencies.

## 5 Discussion

Intraoperative elevation in airway pressure accompanied by hypoxemia constitutes one of the critical situations faced by anesthesiologists. Its etiology is diverse and requires rapid differential diagnosis and management. Common causes primarily include: (1) Mechanical obstruction: such as kinking of the breathing circuit or endotracheal tube, occlusion of the endotracheal tube by sputum plugs, blood clots, or foreign bodies, and inadvertent endobronchial intubation. (2) Airway hyperresponsiveness and bronchospasm [6]: This can be triggered by inadequate anesthetic depth, airway stimulation (e.g., suctioning, tube irritation), anaphylactic drug reactions, or pre‐existing pulmonary conditions (e.g., asthma), manifesting as generalized airway narrowing and a dramatic increase in ventilation resistance. (3) Parenchymal and pleural pathologies [7]: Examples include tension pneumothorax, massive pleural effusion, severe pulmonary edema, or atelectasis, which reduce the effective ventilatory area or restrict lung expansion. (4) Extra‐pulmonary factors [8]: Including diaphragmatic elevation due to pneumoperitoneum during laparoscopic surgery, severe abdominal distension, or surgical positioning that compresses the thoracic cage.

Airway obstruction and severe hypoxemia directly attributable to an ETT cuff malformation are exceedingly rare. Previous studies primarily focus on complications from cuff leaks causing hypoventilation or cuff overinflation leading to cardiac arrest. NagaSawa et al. [9] described a case where cuff pressure remained stable despite continuous air loss. More typical leak cases are iatrogenic, such as pilot balloon injury from towel clip clamping during surgery [5] or cuff tearing during nasotracheal intubation caused by nasal orthodontic micro‐implants [10]. Aron et al. [11] reported a case of hypoxic cardiac arrest secondary to ETT cuff overinflation, where the pathogenic mechanism was excessive external compression of the tracheal mucosa and para‐tracheal structures, rather than obstruction of the ETT lumen itself. The current case differs from all above‐mentioned scenarios in two key aspects: first, the cuff malformation is a latent manufacturing defect rather than an iatrogenic injury or operator error; second, the cuff had no structural rupture. In this case, the pre‐use leak test was normal, and induction and intubation were uneventful, indicating the cuff malformation was a latent defect.

Inspection of the removed ETT revealed a distinct, localized outward protrusion on the anterosuperior aspect of the inflated cuff, disrupting its normal elliptical contour. The Murphy eye, located on the anterior wall near the tube tip, was positioned directly adjacent to this malformed area. The pathophysiological mechanism is postulated as follows: (1) Cuff Wall Weakness & Tube Deviation: A weak area in the cuff wall's right‐superior region caused localized ballooning upon inflation, deviating the tube shaft leftward. This misalignment pressed the distal orifice firmly against the left tracheal wall, obstructing the primary gas flow path (Figure 2). (2) Murphy Eye Occlusion: The abnormal cuff protrusion overlapped and physically occluded the adjacent Murphy eye (the secondary ventilation port). (3) Dual‐Valve Occlusion Effect: Simultaneous obstruction of both the main distal orifice and the Murphy eye created a “dual‐valve occlusion,” preventing effective gas exchange, leading to acute, refractory high airway pressure, and profound hypoxemia.

**FIGURE 2:**
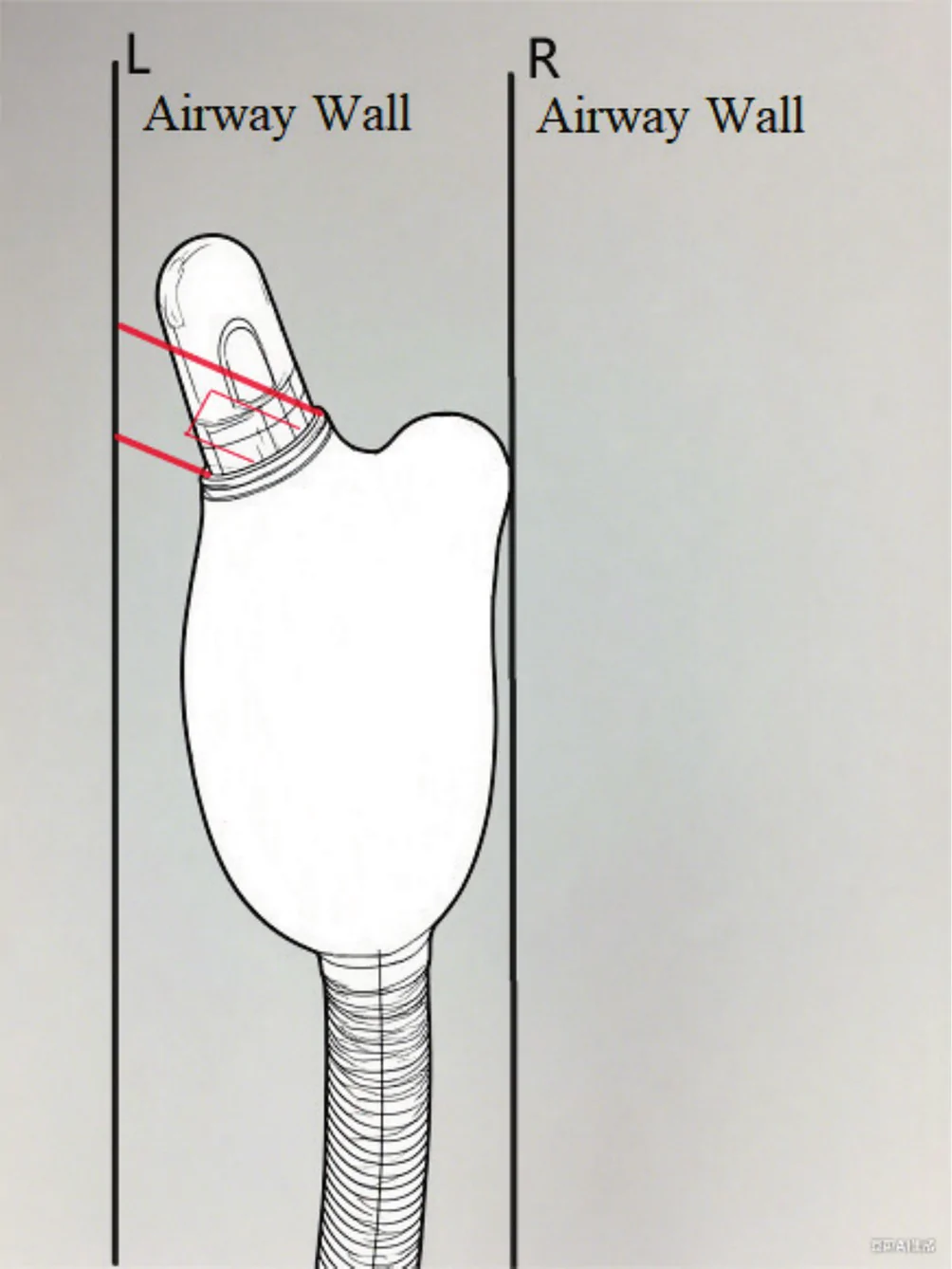
Mechanism of airway obstruction.

Patient repositioning, particularly head and neck movement, can cause ETT migration, potentially resulting in endobronchial intubation. Hartrey et al. demonstrated that even with secure tube fixation, neck movement can cause tube tip migration towards the carina [12]. In this case, the onset of significant airway obstruction and ventilatory difficulty immediately following head repositioning likely involved movement of the malformed cuff into a position where its deformity could occlude the tube orifices. Fiberoptic bronchoscopy is a reliable tool for confirming tube position and inspecting the airways [13]. However, its initially inconclusive findings in this case complicated diagnosis and delayed definitive management. The critical decision to remove the ETT was made when SpO_2_ reached 60%, leading to immediate resolution with mask ventilation and subsequent uneventful re‐intubation.

## Author Contributions

**Xin Li:** data curation, investigation, writing – original draft. **Yuwen Chen:** data curation, formal analysis, investigation. **Dan Zhou:** data curation, formal analysis, writing – review and editing.

## Funding

The authors have nothing to report.

## Consent

Written informed consent from the patient was obtained for publication according to journal guidelines.

## Data Availability

The data that support the findings of this study are available on request from the corresponding author. The data are not publicly available due to privacy.
